# Use of Salivary Diurnal Cortisol as an Outcome Measure in Randomised Controlled Trials: a Systematic Review

**DOI:** 10.1007/s12160-015-9753-9

**Published:** 2016-03-23

**Authors:** Richella Ryan, Sara Booth, Anna Spathis, Sarah Mollart, Angela Clow

**Affiliations:** Palliative Care Department, Cambridge University Hospitals NHS Foundation Trust, Elsworth House, Box 63, Hill’s Road, Cambridge, CB2 0QQ UK; Department of Oncology, University of Cambridge, Hutchison/MRC Research Centre, Cambridge Biomedical Campus, Box 197, Cambridge, CB2 0XZ UK; St. Nicholas Hospice Care, Hardwick Lane, Bury St. Edmunds, Suffolk IP33 2QY UK; Department of Psychology, University of Westminster, 101 New Cavendish Street, London, W1W 6XH UK

**Keywords:** Salivary cortisol, Diurnal rhythm, Randomized controlled trial, Systematic review, Intervention

## Abstract

**Background:**

Dysregulation of the hypothalamic-pituitary-adrenal (HPA) axis is associated with diverse adverse health outcomes, making it an important therapeutic target. Measurement of the diurnal rhythm of cortisol secretion provides a window into this system. At present, no guidelines exist for the optimal use of this biomarker within randomised controlled trials (RCTs).

**Purpose:**

The aim of this study is to describe the ways in which salivary diurnal cortisol has been measured within RCTs of health or behavioural interventions in adults.

**Methods:**

Six electronic databases (up to May 21, 2015) were systematically searched for RCTs which used salivary diurnal cortisol as an outcome measure to evaluate health or behavioural interventions in adults. A narrative synthesis was undertaken of the findings in relation to salivary cortisol methodology and outcomes.

**Results:**

From 78 studies that fulfilled the inclusion criteria, 30 included healthy participants (38.5 %), 27 included patients with physical disease (34.6 %) and 21 included patients with psychiatric disease (26.9 %). Psychological therapies were most commonly evaluated (*n* = 33, 42.3 %). There was substantial heterogeneity across studies in relation to saliva collection protocols and reported cortisol parameters. Only 39 studies (50 %) calculated a rhythm parameter such as the diurnal slope or the cortisol awakening response (CAR). Patterns of change in cortisol parameters were inconsistent both within and across studies and there was low agreement with clinical findings.

**Conclusions:**

Salivary diurnal cortisol is measured inconsistently across RCTs, which is limiting the interpretation of findings within and across studies. This indicates a need for more validation work, along with consensus guidelines.

**Electronic supplementary material:**

The online version of this article (doi:10.1007/s12160-015-9753-9) contains supplementary material, which is available to authorized users.

## Introduction

The hypothalamic-pituitary-adrenal (HPA) axis is known to be an important pathway in the regulation of the physiological stress response. HPA axis dysregulation has been shown to be associated with important health outcomes including psychiatric illness [1], cardiovascular mortality [2], cancer prognosis [3, 4], and frailty and cognitive decline [5]. These associations are thought to be mediated by the deleterious effects of chronic stress on HPA axis function [6], with secondary effects on metabolic, immune and psychobiological systems [7]. The many associations between HPA axis dysregulation and markers of health status suggest that HPA axis modulation by therapeutic interventions may have a role in disease treatment and prevention. In order to demonstrate this, accurate and feasible measurement of HPA axis function within randomised controlled trials (RCTs) is necessary.

The use of salivary cortisol as a biomarker of stress and HPA axis function is a well-established practice in stress research, dating back to at least 20 years [8]. Due to marked diurnal variation in cortisol hormone secretion throughout the day [9], a variety of methods of salivary cortisol collection and analysis have been explored and utilised in an attempt to identify the most representative summary measure of HPA axis function. Essentially, two broad approaches have been taken [9]. The first approach is to measure HPA axis reactivity to a standardised acute stressor. Whilst this approach is useful, interpretation of the results is limited by the need to consider the time of day the stressor is administered, as well as the nature of the stressor. The second approach is to measure basal or unstimulated HPA axis function; thus avoiding the need to administer a stressor.

Measurement of basal HPA axis function has evolved considerably over the past two decades, as theoretical and empirical knowledge regarding the stress system, cortisol measurement and disease associations has increased. In the early days of salivary cortisol research, a single salivary cortisol measure, collected at a pre-specified time, was used to estimate basal HPA axis function, but this methodology proved to be unreliable, with large intra-individual and inter-individual variation [10]. Another common approach was to measure average or total cortisol exposure over a 12–24-h period [11]. Whilst this approach provides a summary measure, it does not accommodate the complex nature of HPA axis aberration, with both hypocortisolism and hypercortisolism now recognised to be linked to chronic stress [12]. Increasingly, therefore, there has been a move towards measuring the circadian rhythm of diurnal cortisol secretion, rather than focusing on absolute cortisol concentration [11].

Typically, under basal conditions, a healthy HPA axis is characterised by a distinctive circadian pattern of cortisol secretion, whereby cortisol rises to a peak within 30–45 min of waking and then falls to a nadir during sleep at approximately midnight [9, 11]. The major measurable parameters of this diurnal rhythm are (1) the cortisol awakening response (CAR), which is the rise in cortisol during the first 30–45 min following awakening [9], and (2) the diurnal cortisol slope, which is the rate of decline in cortisol levels across the day, from morning to evening [11]. This normal rhythm becomes disrupted when the HPA axis becomes dysregulated [9, 13], the pattern of disruption varying depending on the context or condition studied. In general, an abnormal cortisol awakening response, both abnormally large and small, or a flattened diurnal cortisol slope appear to be consistent markers of HPA axis dysfunction [11]. Importantly, there is evidence that these parameters are independently regulated, with the cortisol awakening response being mediated by an extra-pituitary pathway to the adrenal from the suprachiasmatic nucleus [14]. Thus, these parameters are believed to represent different aspects of HPA axis function [14–16].

Due to the many possible ways of measuring salivary cortisol as a biomarker in stress research, it is necessary to reach consensus regarding the most appropriate methodology, so that the results of different research studies can be compared and so as to avoid waste in the design, conduct and reporting of studies. Adam and Kumari [11] have reviewed the use of salivary diurnal cortisol in epidemiological studies and have, accordingly, published recommendations. They found that the cortisol awakening response, the cortisol slope and the area under the daytime cortisol curve (AUC) were most commonly measured within large epidemiological studies and have been most robustly linked with psychosocial phenomena and health outcomes, implying clinical relevance. They recommend that these parameters should each be assessed as separate indicators of HPA axis function and that the cortisol collection schedule be sufficient to measure, at minimum, the cortisol awakening response and the diurnal slope over more than 1 day.

There is no guidance available for the measurement of salivary diurnal cortisol within interventional studies, and little is known about how salivary cortisol has been employed, to date, as a biomarker within RCTs of health and behavioural interventions. The inherent complexity of salivary diurnal cortisol as a biomarker is likely to pose particular challenges within RCTs. Given that the diurnal profile is essentially a composite of at least three measurement parameters (the cortisol awakening response, diurnal slope and area under the curve), each reflecting different aspects of HPA axis function, it is possible that experimental interventions will have different effects on different parameters. This is likely to impact on a priori decisions about the primary measurement parameter of interest, on hypotheses about directions of change and on conclusions about efficacy, target engagement and mechanisms of action. As well as these challenges, there are concerns in the literature about the long-term stability of this biomarker over periods of greater than 1 month [17], as well as concerns about its reliability in the shorter term due to day-to-day state effects [18] and the effects of non-compliance [19]. Concerns have also been raised about the responsiveness of the biomarker and how different contexts and populations may impact on this [20].

To assess whether specific guidance is necessary, we systematically reviewed the literature with the aim of describing the RCTs of health and behavioural interventions which have used salivary diurnal cortisol as an outcome measure, particularly focusing on salivary diurnal cortisol methodology and findings. Specifically, we aimed to explore the following questions:

1. Which health and behavioural interventions have been evaluated using salivary diurnal cortisol?
2. What populations have been evaluated?
3. What collection protocols have been used to obtain a diurnal cortisol profile?
4. What parameters of the diurnal cortisol profile have been measured?
5. Where a change in a cortisol profile parameter is observed, when in the follow-up period does it occur?
6. How often is there consistency between the clinical and cortisol response to the intervention?

## Methods

The protocol for this review is available in the Electronic Supplementary Material 1.

### Study Inclusion and Exclusion Criteria

We restricted the sample to RCTs only. Though the review question is relevant to non-randomised uncontrolled longitudinal studies also, we chose to select RCTs only in order to reduce the scope of the search and the heterogeneity of the sample; this was deemed necessary given the broad review question with respect to interventions and sample populations. We also expected that RCTs would be of higher quality than other study designs, thus providing more reliable information. We defined ‘diurnal cortisol profile measurement’ as the collection of at least two samples of salivary cortisol over at least 1 day. This would enable the calculation of the diurnal slope or the cortisol awakening response, at minimum. Within the context of an RCT, there needed to be evidence that this measurement had been obtained on at least one occasion before the intervention and on at least one separate occasion (i.e. a separate day or period of days) after the intervention. Using these definitions, we adhered to the following inclusion and exclusion criteria:

#### Inclusion Criteria

1. Population: any adult (>18 years) population.
2. Study design: randomised controlled trials.
3. Interventions: any type of therapeutic intervention designed to improve an aspect of health or well-being, excluding exogenous corticosteroids.
4. Control or comparator: any type of control or comparator.
5. Outcome measures: studies that use salivary diurnal cortisol profile measurement as a primary or secondary outcome measure.

#### Exclusion Criteria

1. Non-RCT studies, including quasi-randomised controlled trials and trial protocol reports without results
2. Studies which use non-diurnal salivary sampling methods e.g. single salivary cortisol measures pre and post an intervention or salivary cortisol pre and post a stress-task
3. Studies which evaluate the effects of exogenous glucocorticoids (any type or route) on salivary cortisol
4. Studies which evaluate the cortisol response to stress-inducing interventions or conditions
5. Studies which measure diurnal cortisol under laboratory-induced conditions (e.g. light-wake conditions)
6. Studies in which the diurnal profile is not measured both before and after the intervention
7. Studies in which the diurnal profile is obtained on the same day as the intervention with a view to assessing its acute effects within the day
8. Studies in people with Cushing’s disease
9. Animal studies
10. Abstract publication available only
11. Dissertation or non-journal publication available only
12. Non-English language publications

### Search Methods for Identification of Studies

On 21 May 2015, we searched the following electronic databases using NHS Evidence Healthcare Databases Advanced Search tool: MEDLINE (1980 to May 2015), CINAHL (1980 to May 2015), PsychINFO (1806 to May 2015), AMED (1985 to May 2015), EMBASE (1974 to May 2015) and the Cochrane Central Register of Controlled Trials. We sought to identify a combination of keywords and MESH terms in the titles and abstracts of papers, adapting the search strategy, as appropriate, for each database. By way of example, the following keywords, MESH terms and publication types were searched in MEDLINE: [(‘cortisol’ AND ‘saliva*’) OR (HYDROCORTISONE/ AND SALIVA/)] AND [(‘randomized controlled trial’ OR ‘controlled clinical trial’ OR ‘randomized’ OR ‘placebo’ OR ‘randomly’ or ‘trial’ OR ‘groups’, excluding ANIMALS (exploded term)]. See Appendix A for more detailed search strategies, including strategies used in the other databases.

### Study Selection for Inclusion in the Review

All abstracts generated from the electronic searches were exported to Endnote X3 for removal of duplicates. One review author (RR) screened the titles and abstracts for the eligibility criteria, and where eligibility could not be determined, the full-text article was obtained. All full-text articles were reviewed for eligibility by RR, and a random selection of these articles (37 %) were reviewed independently by the other four authors (SB, AS, AC and SM), each reviewing a different selection, to ensure that the eligibility criteria were being correctly interpreted and adhered to. The inclusion criteria were applied in a hierarchical manner, first checking the population, then the study design, then the intervention and finally the cortisol methodology. Any disagreement was discussed in the first instance between the two authors in question. If consensus was not achieved between the two authors in question, a third party (one of the other authors) was consulted.

### Data Collection and Extraction

All full-text articles which were deemed eligible were reviewed in more detail for data extraction. RR completed the data extraction for all eligible texts. In addition, another author independently completed data extraction on 10 % of the eligible articles to ensure that data were being extracted appropriately. A data extraction form containing the following fields was used to summarise the pertinent details of the study: trial ID, eligibility criteria checklist with decision outcome, study design, population, intervention/control, salivary cortisol collection protocol details, other outcome measures, salivary cortisol analysis details, salivary cortisol results and clinical outcome results.

### Assessment of Quality and Relevance

The quality of each study and its relevance to the review aim were assessed using the Gough Weight of Evidence framework [21], which uses four domains of assessment (A, B, C and D), rating each domain as low, moderate or high. As the overarching aim of this review was to describe salivary cortisol methodology and findings in RCTs, the relevance of each study was assessed purely in relation to the degree to which it contributed information towards this aim.

Within the first domain of this framework, judgments are made in relation to the generic quality of execution of the study independent of the review aim (Weight of Evidence A). Within the second and third domains, judgements are made in relation to the specific aim of the review, including the appropriateness of the study design to the review aim (Weight of Evidence B) and the focus of the study, including its objectives and reporting, relative to the review aim (Weight of Evidence C). An overall judgement of quality and relevance (low, moderate or high) is formed by combining the assessments for these domains (Weight of Evidence D). This framework is weighted more heavily towards relevance than quality and was chosen as a means of highlighting those studies which provided the most relevant information towards the review aim. This was deemed to be the most appropriate approach to appraising the literature included in this review, given that we were not concerned with evaluating the efficacy of specific interventions.

### Data Synthesis and Presentation

The selection process is presented using a PRISMA flow chart. A narrative synthesis of the scope, characteristics and findings of the selected studies is given and presented in tables. To enhance clarity and facilitate comparison, individual studies were organised into four categories according to the intervention being evaluated, and a separate table of studies was created for each category. Counts and percentages were used to describe data patterns across all studies and between study intervention categories. Medians and interquartile ranges were calculated to describe key features of the salivary collection protocol across studies and to describe the frequency and distribution of the follow-up time-points at which cortisol findings occurred. For a cortisol parameter, a significant finding was considered to be present if a study reported a statistically significant within- or between-group effect from baseline to follow-up, for either the intervention or the comparator group. If a significant finding was also reported for at least one clinical outcome measure in the same study, using the same statistical tests, the cortisol findings were considered to support the clinical outcome. In the same way, if there was no evidence of a statistically significant effect for both the cortisol and clinical outcomes, cortisol findings were considered to support clinical findings.

## Results

### Selection Process

**Fig. 1:**
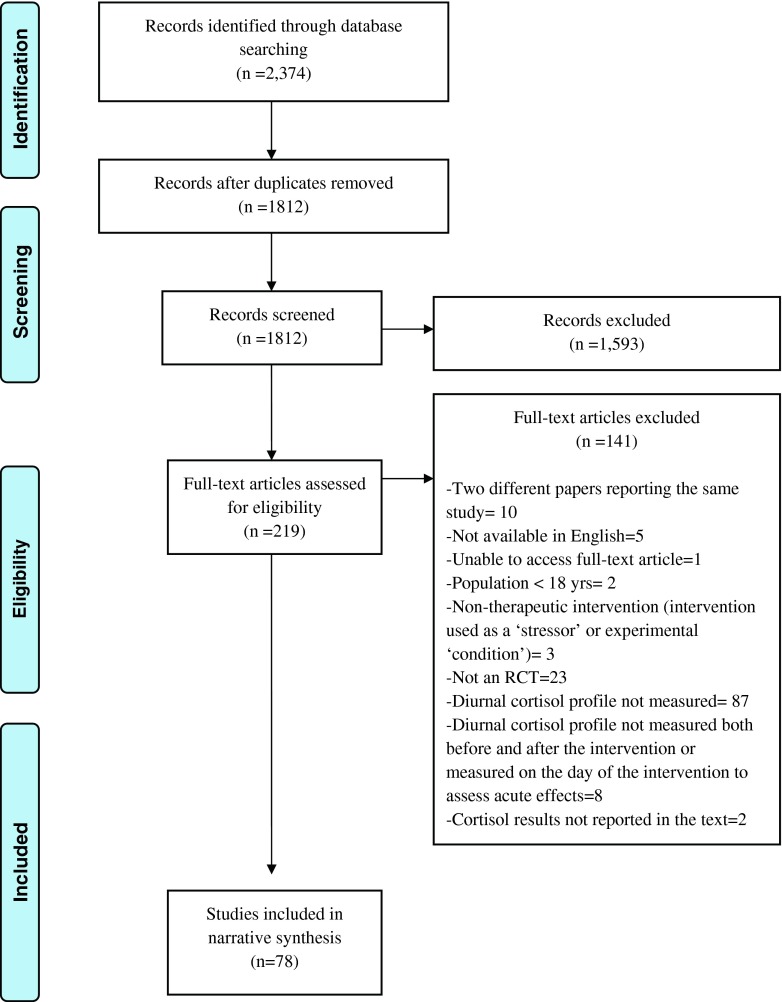
PRISMA flow diagram illustrating the identification of studies

The process of screening and reviewing articles for eligibility is summarised in Fig. 1. The database search identified 2374 articles. After removal of duplicates using Endnote X3, 1812 potentially relevant abstracts were identified. Screening of these abstracts led to the selection of 219 full-text articles for more detailed eligibility assessment. Of these full-text articles, 78 studies were selected for inclusion in the review after removal of ineligible studies and after exclusion of duplicate reports of the same study. The most common reason for exclusion of articles related to salivary cortisol methodology. After excluding studies due to ineligible populations, interventions and designs, 87 of the remaining 175 RCTs (50 %) were excluded because the salivary cortisol measurements therein did not allow analysis of the diurnal rhythm. In most cases, this was due to the measurement of cortisol on a single occasion before and after the intervention being evaluated. A small number of RCTs (*n* = 10), which did include diurnal cortisol measurements, were later excluded from the review because their cortisol findings were not adequately reported or because their measurements were conducted in a way that was not comparable with the other studies.

### Characteristics of Included Studies

**Fig. 2:**
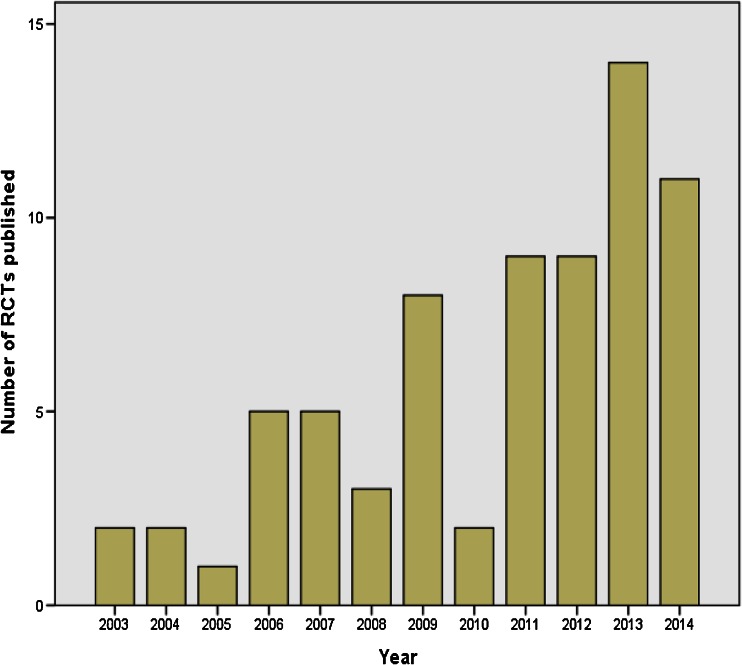
Number of eligible RCTs published per year. The year 2015 was excluded from this graph as the complete results for this year are not yet available

Included studies were published from 2003 to May 2015. There has been a notable increase in the number of published RCTs using salivary diurnal cortisol as an outcome measure in the past decade, with the yearly rate increasing from 2 studies per year in 2003 and 2004 to 14 studies in 2013 and 11 studies in 2014 (see Fig. 2). Indeed, over 50 % of the included studies have been published since 2012. Pertinent characteristics of individual studies are presented within Tables 1, 2, 3 and 4, with each table representing one of four study categories and each study being organised into one of such categories according to the intervention being evaluated: (1) RCTs evaluating psychosocial interventions, (2) RCTs evaluating pharmacological (including nutritional) interventions, (3) RCTs evaluating complementary therapies and (4) RCTs evaluating all other types of interventions.

**Table 1:**
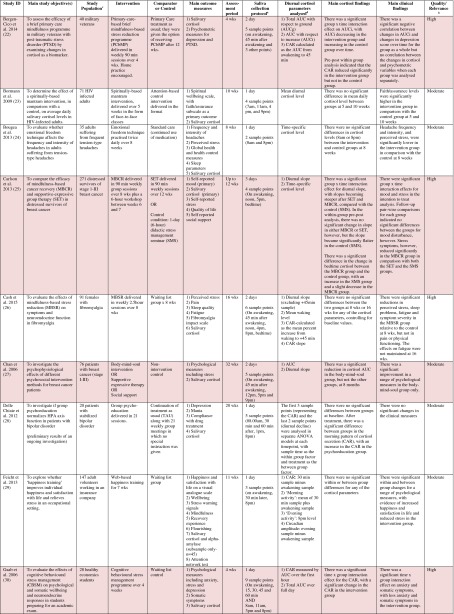

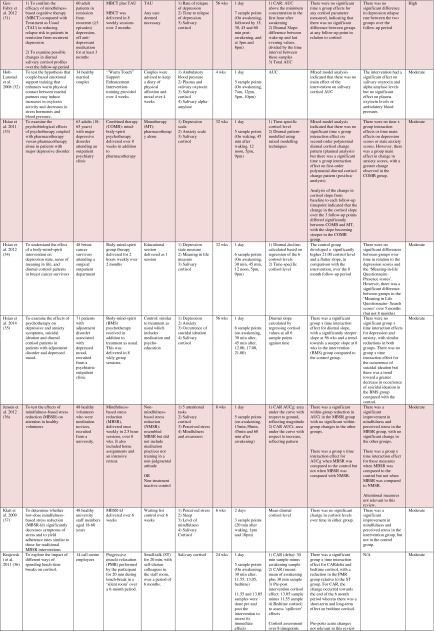

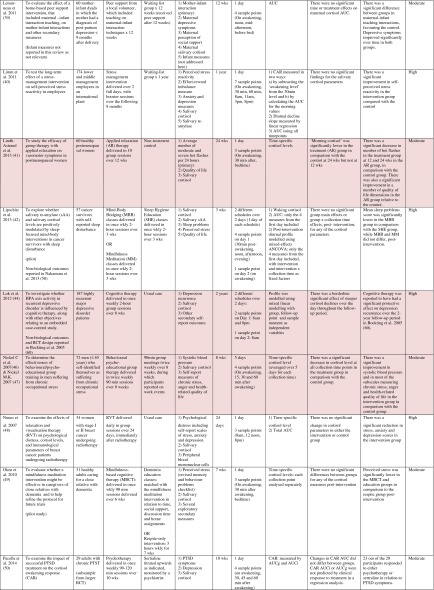

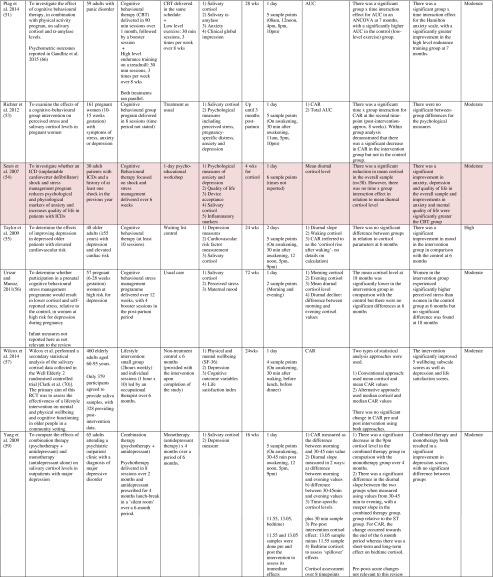
Randomised controlled trials evaluating psychosocial interventions: study characteristics, salivary cortisol methodology and main findings

Shaded rows represent studies with agreement between clinical and cortisol findings

^a^Describes the number of consecutive days of sampling and the number of samples per day per time-point

^b^Assessed using Gough’s Framework

^c^Refers to the number and type of participants randomised

^d^Abbreviations used for cortisol summary measures: CAR, cortisol awakening response; AUC, area under the curve.

**Table 2:**
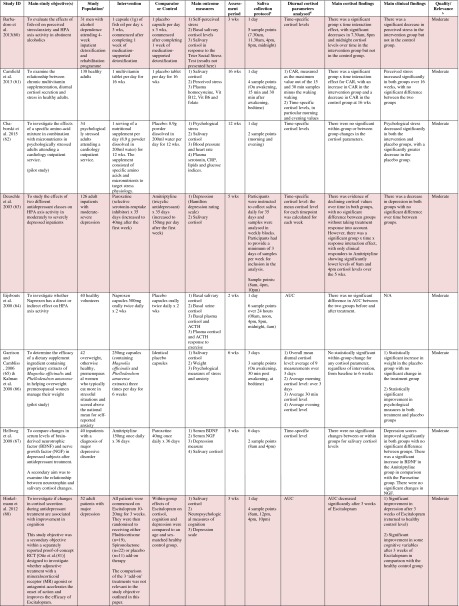

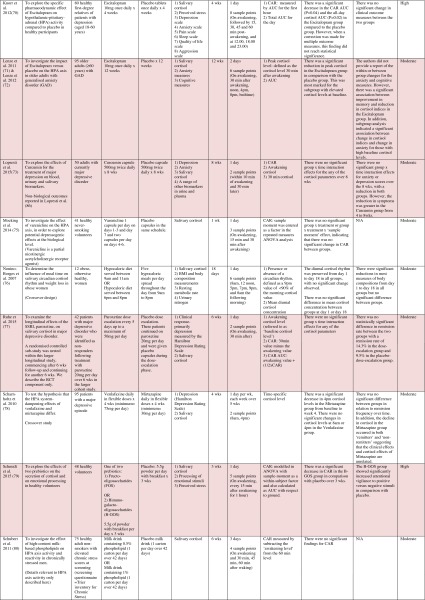

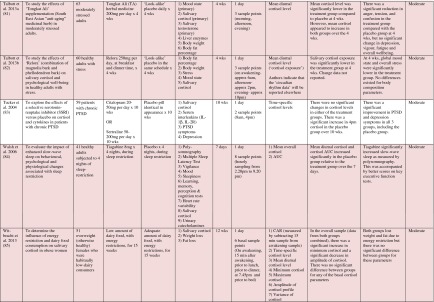
Randomised controlled trials evaluating pharmacological (including dietary) interventions: study characteristics, salivary cortisol methodology and main findings

Shaded rows represent studies with agreement between clinical and cortisol findings

^a^Describes the number of consecutive days of sampling and the number of samples per day per time-point

^b^Assessed using Gough’s Framework

^c^Refers to the number and type of participants randomised

^d^Abbreviations used for cortisol summary measures: CAR, cortisol awakening response; AUC, area under the curve.

**Table 3:**
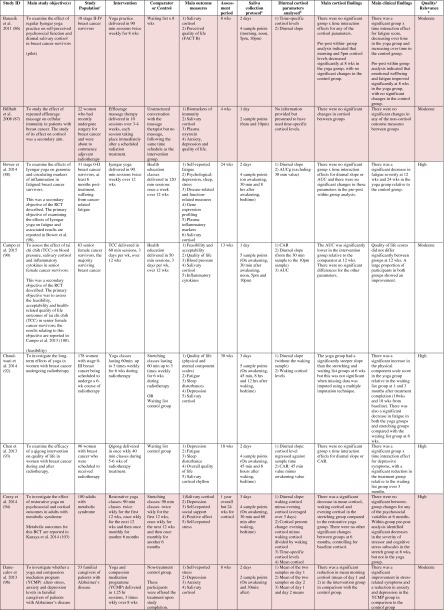

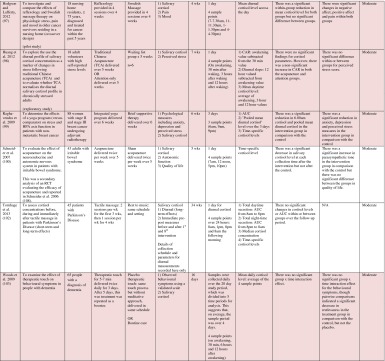
Randomised controlled trials evaluating complementary therapies: study characteristics, salivary cortisol methodology and main findings

Shaded rows represent studies with agreement between clinical and cortisol findings

^a^Describes the number of consecutive days of sampling and the number of samples per day per time-point

^b^Assessed using Gough’s Framework

^c^Refers to the number and type of participants randomised

^d^Abbreviations used for cortisol summary measures: CAR, cortisol awakening response; AUC, area under the curve.

**Table 4:**
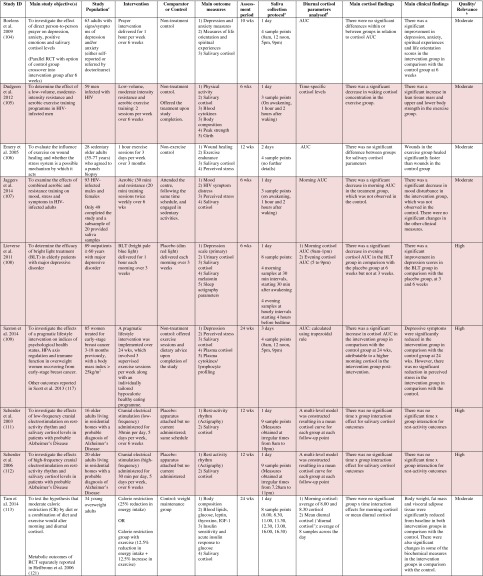
Randomised controlled trials evaluating a range of ‘other’ interventions: study characteristics, salivary cortisol methodology and main findings

Shaded rows represent studies with agreement between clinical and cortisol findings

^a^Describes the number of consecutive days of sampling and the number of samples per day per time-point

^b^Assessed using Gough’s Framework

^c^Refers to the number and type of participants randomised

^d^Abbreviations used for cortisol summary measures: CAR, cortisol awakening response; AUC, area under the curve.

Most commonly, studies evaluated psychosocial interventions (*n* = 33, 42.3 %), such as cognitive behavioural therapy, mindfulness and psychotherapy (see Table 1). Pharmacological therapies, including nutritional therapies, comprised the second most common intervention category (*n* = 22, 28.2 %), within which eight studies evaluated anti-depressant medications (see Table 2). Complementary therapies were evaluated in 14 studies (17.9 %), with six of these studies evaluating yoga (see Table 3). Nine studies evaluated treatments which did not fall into the three major categories and were, therefore, classified as ‘other’ (see Table 4). These treatments included exercise (*n* = 3), cranial electrostimulation (*n* = 2), a lifestyle intervention (*n* = 1), dietary restriction (*n* = 1), prayer (*n* = 1) and light treatment (*n* = 1).

Interventions were evaluated in a wide variety of study populations. Overall, across all studies, these populations could be broadly classified as follows: people who were healthy or at risk of disease (*n* = 30, 38.5 %), patients with physical or psychosomatic disease (*n* = 27, 34.6 %) and patients with a psychiatric diagnosis (*n* = 21, 26.9 %). Physical and psychosomatic disease categories included current or prior cancer (*n* = 14; predominantly breast cancer), cardiovascular disease or metabolic syndrome (*n* = 3), human immunodeficiency virus (*n* = 3), dementia (*n* = 3), Parkinson’s disease (*n* = 1), irritable bowel syndrome (*n* = 1), tension headache (*n* = 1) and fibromyalgia (*n* = 1). Psychiatric pathology included depression (*n* = 12), anxiety disorder (*n* = 2), co-morbid anxiety and depression (*n* = 1), post-traumatic stress disorder (*n* = 3), adjustment disorder (*n* = 1), bipolar disorder (*n* = 1) and alcoholism (*n* = 1). Within study categories, psychosocial intervention studies and pharmacological studies most commonly evaluated people who were healthy or at risk of disease (42 and 54.5 % of psychosocial and pharmacological studies, respectively), whereas complementary therapy studies most commonly evaluated patients with physical or psychosomatic disease (86 % of complementary therapy studies).

Overall, across study categories, the median study sample size was 55 participants (IQR 34–77), with the smallest study including only 12 participants [76] and the largest including 379 participants [57]. Median sample size was similar between study categories: 59 (IQR 34–74) for psychosocial interventions, 51 (IQR 41–76) for pharmacological interventions, 49 (IQR 21–90) for complementary therapies and 59 (IQR 24–87) for other interventions. Eight studies were reported as pilot, feasibility or exploratory studies. Overall, the median length of follow-up from baseline was 10 weeks (IQR 4 to 21) and ranged from 1 to 72 weeks.

Quality and relevance (aggregate score) were rated as high for 20 studies and moderate for 58 studies. No study was given an aggregate score of low, reflecting the exclusion of studies of low relevance by the eligibility criteria. Of note, many studies were of low quality with respect to their RCT design but of high relevance with respect to the review aim, resulting in a high overall aggregate score using Gough’s framework [21]. See Appendix B for the breakdown of scores per domain for each included study.

### Salivary Cortisol Collection and Analysis Methodology

Pertinent details relating to the salivary cortisol collection protocols and parameters used in individual studies are presented in Tables 1, 2, 3 and 4.

In relation to saliva collection, the median number of days of saliva collection per time-point across studies was 1 day (IQR 1–2). As the median suggests, the majority of studies (*n* = 57, 73.1 %) collected saliva over 1 day only. Ten studies used protocols of 2 days, eight used protocols of 3 days and three used protocols of 4–6 days. The median number of samples collected per day was 4 (IQR 3–5), ranging from a minimum of two samples per day (*n* = 12) to a maximum of nine samples per day (*n* = 3). Seventeen studies (21.8 %) collected six or more samples per day. There was substantial variation in the timings of the samples per day. Importantly, of the 75 studies which reported sample times, only 49 (62.8 %) included an awakening sample, suggesting that many studies did not use waking time as a reference for subsequent diurnal sampling points, choosing clock times in preference.

A wide range of different salivary diurnal cortisol parameters were analysed both within and across studies. In relation to composite measures of diurnal cortisol, the cortisol awakening response was measured in 25 studies (32.1 %), the area under the curve from morning to evening was measured in 22 studies (28.2 %) and the diurnal decline/slope was measured in 18 studies (23.1 %). Five studies (6.4 %) modelled the diurnal profile using multi-level modelling techniques, obviating the need to calculate these composites separately. It was also common for studies to report on changes in mean cortisol levels across the day (18, 23.1 %) or changes in absolute cortisol levels as specific times during the day (34, 43.7 %). The majority of studies (42, 53.8 %) used just one of these methods of analysing and reporting diurnal cortisol, but 25 studies (32.1 %) used two methods and 11 studies (14.1 %) used three or more methods.

Overall, 39 studies (50 %) measured an indicator of circadian rhythm by calculating either a cortisol awakening response or a diurnal slope or by modelling the diurnal profile. Interestingly, this proportion differed between study intervention categories: psychosocial intervention studies (22/33, 66.7 %), pharmacological studies (8/22, 36.4 %) and complementary therapy studies (7/14, 50 %). Only nine studies (11.5 %) calculated both the cortisol awakening response and the diurnal slope. In addition, however, five studies modelled these simultaneously using multi-level modelling techniques, suggesting that 14 studies in total (17.9 %) analysed both indicators of HPA axis regulation. Only two studies (2.6 %) used all three diurnal profile parameters (cortisol awakening response, diurnal slope and area under the curve) as recommended by Adam et al. for epidemiological studies [11].

### Cortisol Findings: Overall Patterns Across Studies and Patterns Within Intervention Categories

Of the 78 included studies, 40 (51.2 %) reported a significant within- or between-group difference in at least one cortisol parameter in response to the experimental intervention or comparator. The significant effects for cortisol parameters were reported at a variety of different follow-up time-points from baseline, ranging from 1 week [38] to 72 weeks [56]. Fifty per cent occurred at a median of 6 weeks from baseline (IQR 4–12).

Seventy-four studies reported both cortisol and clinical findings, and these findings were in agreement in only 50 % of cases; these studies are shaded in Tables 1, 2, 3 and 4. The rate of agreement between clinical and cortisol findings differed between study intervention categories: psychosocial interventions (11/32, 34.4 %), pharmacological studies (11/20, 55 %) and complementary therapy studies (9/13, 69 %). In most cases of disagreement, significant effects for clinical outcome measures were not accompanied by significant effects for cortisol measures (25/37, 67.5 %). In some cases, significant effects were found for cortisol measures without significant effects for clinical measures (10/37, 27 %), and in two cases, significant effects occurred at different time-points for the two types of measures.

As expected, due to wide heterogeneity across studies in multiple domains (e.g. interventions, populations and cortisol parameters), it was not possible or meaningful to summarise the cortisol findings across all studies. Therefore, as an example of the type and range of findings reported, we chose to compare and contrast the cortisol outcomes for one study population, the breast cancer population, following similar interventions. This population was chosen as a number of larger RCTs have been conducted in this population in recent years, with many scoring ‘high’ in the quality and relevance assessment. In addition, there is robust evidence that flatter diurnal cortisol slopes in this population are associated with shorter survival [3], pointing to the plausibility of the HPA axis as a potential therapeutic target.

Four studies evaluated the effects of different psychosocial interventions in patients with breast cancer. There was inconsistency between clinical and cortisol findings in two of these studies. In addition, the types of cortisol parameters measured, along with their patterns of change, were not uniform across studies. Two studies, one evaluating mindfulness-based cancer recovery and supportive expressive therapy [25], and the other evaluating mind-body-spirit therapy [34], found that the diurnal slope remained unchanged in the treatments groups but that it became significantly flatter in the control group, suggesting that these treatments had a buffering effect on the HPA axis. This finding corresponded with clinical findings in only one of the studies [25], however. In another study evaluating mind-body-spirit therapy, there was no change in the diurnal slope in either the treatment or the control groups, but the area under the curve decreased in the treatment group, mirroring a reduction in symptoms in this group [27]. A similar intervention (relaxation and visualisation therapy) had no effect on the area under the curve in another study, however, despite a reduction in symptoms [48].

Four studies evaluated yoga in patients with prior or current breast cancer. Banasik et al. [86] found no significant change in the diurnal slope, despite an improvement in symptoms; however, absolute cortisol levels (morning and evening) were found to be significantly reduced. Bower et al. [88] found that there was no change in the diurnal slope or the area under the curve, despite an improvement in symptoms. A further study found that, along with symptom improvement, the diurnal slope became significantly steeper over 6 weeks of treatment relative to comparator groups; this finding lost significance, however, after missing values were addressed using a multiple imputation technique [92]. Finally, Raghavendra et al. [99] found a reduction in symptoms, 6.00 am cortisol concentration and ‘pooled mean diurnal cortisol’ after yoga but no change in other time-specific cortisol levels or in the area under the curve. All four of these studies provide support for yoga in relation to symptom improvement, but there was no consistent pattern of change in cortisol parameters across studies. The problem of interpreting cortisol findings was further compounded by the use of a range of different cortisol parameters both within and between studies.

## Discussion

This systematic review characterises the types of RCTs which have used salivary diurnal cortisol as an outcome measure for the evaluation of health and behavioural interventions and details the salivary diurnal cortisol methodology and findings therein. To the authors’ knowledge, this is the first systematic review of this kind.

The review highlights the increasing use of salivary diurnal cortisol as an outcome measure in RCTs, particularly since 2012. The majority of these RCTs have evaluated psychosocial or complementary therapy interventions in a wide range of populations, ranging from healthy volunteers to patients with cancer. With regard to salivary diurnal cortisol methodology and outcomes, the review has identified the following findings: (1) many of the RCTs screened did not use diurnal measures of salivary cortisol, (2) the majority of RCTs measuring diurnal cortisol collected samples over 1 day only, (3) there is wide heterogeneity across studies in relation to sampling schedules, (4) there is wide heterogeneity in relation to the cortisol profile parameter chosen for analysis, with a large proportion of studies failing to analyse diurnal rhythm parameters, and (5) interpretation of cortisol findings within and between RCTs is challenging due to the use of different parameters in different studies, varying cortisol change patterns across studies and high levels of inconsistency between cortisol and clinical findings. These review findings are discussed below, and based on these findings, recommendations are made for the future incorporation of salivary diurnal cortisol into RCTs.

### Many of the RCTs Screened Did Not Measure Diurnal Cortisol Profiles

During the selection process, after excluding articles for other reasons, 87 of the remaining 175 RCTs (50 %) were excluded because they did not measure diurnal profiles of salivary cortisol, despite including it as an outcome measure. In many cases, a single salivary cortisol sample was obtained before and after an intervention, either on the same day as the intervention was received or on a different day. It is long established that single measures of basal cortisol, even if collected at the same time each day, have very low reliability due to significant intra-individual variability [10]. For example, Coste et al. [115] demonstrated that when a single salivary cortisol sample was collected at 8 am at three time-points over 5 weeks the intra-class correlation coefficient (*r*) was as low as 0.18. In addition, single measures of basal cortisol have very low diagnostic utility, due to wide inter-individual variation, with normal ranges overlapping with abnormal ranges [10]. It is surprising that despite this knowledge, which dates back to 1994, many researchers are still using single measures of cortisol as biomarkers within their trials. This practice has the potential consequence of generating false positive results in response to interventions, particularly within small pilot studies. Apart from this, the use of unreliable measures within RCTs is a waste of limited financial resources.

### The Majority of RCTs Collected Saliva Samples Over 1 Day Only

This review found that 57 out of the 78 included studies (73.1 %) collected diurnal samples over 1 day only. When the cortisol awakening response is measured on a single day, it has been shown to be highly influenced by situational or state factors, but reliable cortisol awakening response measurements have been obtained when the cortisol awakening response is averaged over at least 2 days [correlation coefficient (*r*) between 2-day pairs = 0.7] [116]. Significant day-to-day variation has also been observed for the diurnal slope, where the frequencies of inconsistent diurnal patterns over 2–3 days were observed to be 31 % in one sample [117] and 43 % in another [118]. For these reasons, it is recommended that salivary cortisol is collected over more than 1 day in order to capture stable characteristics [11]. In fact, it has been suggested that it is better to add more consecutive days to the protocol than more samples per day in order to improve the reliability of diurnal rhythm assessment [11]. Measures of low reliability inevitably result in low validity. Therefore, the predominant lack of consecutive day sampling observed in this review necessitates that cortisol outcomes within the included RCTs be interpreted with caution. Indeed, the low level of agreement between cortisol and clinical findings across the RCTs (50  %) might well be explained by the low reliability of the diurnal profiles measured within these RCTs.

### There Is Wide Heterogeneity Across Studies in Relation to Sampling Schedules

Within the included studies, the number of samples collected per day ranged from two samples per day to nine samples per day, the median being 4 (IQR 3–5) samples per day. The wide variation in protocols highlights the fact that there really is no consensus regarding the optimal frequency of sampling per day. Some of this is probably due to lack of knowledge in the field of stress research about the impact of different sampling schedules on diurnal profile validity. In their review, Adam and Kumari [11] referred to unpublished data of theirs which demonstrated that a 2-point diurnal slope (morning and evening) correlates extremely well with a 6–7 point slope (correlation coefficient = 0.94), suggesting that delineating the curve more precisely does not significantly improve the accuracy of important summary measures such as the diurnal slope. Whilst this data suggests that a minimal protocol of 2 collection points per day can yield a meaningful diurnal slope, further validation studies are needed to confirm this and to investigate the maximum number of samples per day beyond which sampling would be wasteful and unnecessarily burdensome. Considering that 21.8 % of RCTs in this review used a schedule of 6 or more sampling points per day, this area of uncertainty needs to be addressed promptly.

In addition to variation in sample number per day, sampling times also differed between studies. For example, only 62.8 % of studies included an awakening sample. As a result, in many studies, the cortisol profile was anchored to clock time rather than waking time, which is suboptimal practice. Whilst it is preferable to calculate the diurnal slope using values outside of the awakening period, the cortisol profile from which it is derived should be anchored to waking time. The rationale for this is well documented, the practice being based upon the fact that waking up activates a burst of cortisol pulses which serve to ‘synchronise’ the circadian rhythm of the HPA axis [10]. Furthermore, it has been shown that diurnal cortisol rhythms are influenced primarily by personal sleep-wake cycles, predominantly wake time, rather than by dark-light cycles [11, 119].

### There Is Wide Heterogeneity Across RCTs in Relation to the Cortisol Profile Parameters Analysed

Despite rhythm parameters being most robustly linked with health outcomes, it was surprising that only half of the RCTs included a marker of diurnal rhythm by measuring either the cortisol awakening response or the diurnal slope or by multi-level modelling techniques. Interestingly, these measures were most commonly used in studies measuring psychosocial interventions where the prevalence was 66.7 %. Lack of measurement of these parameters within RCTs suggests little awareness of the complexities of HPA axis regulation and function amongst clinical trialists and points to the need to better translate psychoneuroendocrinological knowledge into clinical trials research. Better collaboration between basic scientists, in the field of psychoneuroendocrinology, and clinical trialists, with an interest in salivary cortisol as a biomarker, may help ameliorate this problem. The higher prevalence of rhythm parameters in psychosocial intervention studies probably reflects the already well-established relationship between the disciplines of clinical psychology and psychoneuroendocrinology, owing to the natural proximity of the fields.

It was uncommon for RCTs using rhythm parameters to measure both the cortisol awakening response and the diurnal decline (17.9 %) and even more uncommon for RCTs to measure all three parameters recommended by Adam and Kumari [11] in epidemiological studies (the cortisol awakening response, diurnal decline and area under the curve) (2.6 %). In the context of RCTs, it would appear sensible to measure all three parameters in order to robustly assess HPA axis activity, particularly in the context of an exploratory study. In particular, it would make sense to measure both the cortisol awakening response and the diurnal slope given that they are believed to be regulated independently, representing different aspects of HPA axis function [14–16, 120]. Failure to measure all parameters within an RCT may result in false negative findings in relation to HPA axis function and may partially explain the low agreement between cortisol and clinical findings in this review. On the other hand, where all three parameters are used, it would be important to guard against the practice of multiple testing and post hoc hypotheses. With this in mind, it would be wise for RCTs to state the primary HPA axis parameter of interest, including its hypothesised direction of change, in the protocol prior to commencing the study.

Studies which did not use rhythm parameters relied on ‘area under the curve’ measures, mean diurnal cortisol measures or absolute cortisol measures at specific times of the day to measure HPA axis activity. There are several disadvantages to these approaches. In relation to the area under the curve, whilst it is a useful measure of overall cortisol exposure, it is difficult to interpret its meaning without a co-measure of diurnal rhythm. This is because both hypocortisolism and hypercortisolism have been linked with chronic stress and its health implications [6], such that the amount of cortisol in the system has become a less discerning instrument for measuring clinically relevant stress. For the same reason, measurement of the mean cortisol level across the day has similar limitations. The measurement of absolute cortisol levels at specific times in the day and the reporting of within- or between-group pre-post changes for each specific time represented another approach. Due to the separate analysis for each sample point, however, this is no different, in many respects, to obtaining multiple single cortisol measures, with each cortisol measure having low reliability. In addition, with this approach, study findings are likely to become contaminated by false positive findings due to the inevitable consequences of multiple analyses. Thus, the RCTs which used this approach need to be interpreted with caution.

### Interpretation of Cortisol Findings Within and Between RCTs Is Challenging

Analysis of the cortisol findings for psychosocial intervention studies and complementary therapy studies in the breast cancer population demonstrated the challenge of interpreting cortisol findings both within and between RCTs. This population, as a whole, is believed to have a flatter diurnal slope than a healthy population, and assuming this relates to chronic stress, one would expect a stress-relieving intervention to result in a steeper slope. No study was able to robustly demonstrate this, however. Instead, the findings of two studies [25, 34] suggested that the diurnal slopes would have become progressively flatter without intervention, due to a pattern of progressively flattening slopes in the control groups. In the absence of longitudinal studies of HPA axis regulation over weeks, months and years, it is not possible to firmly draw this conclusion, however. The findings of these studies illustrate the importance of understanding the natural history of HPA axis regulation within the target population before evaluating the effects of interventions in RCTs. Without understanding this, it is not possible to form a priori hypotheses regarding the direction of change in a cortisol parameter in response to an intervention. It may well be that stress-relieving interventions serve to ‘stabilise’ the HPA axis and protect it from further dysregulation, but this can only occur in a population within which unstable function or progressive HPA axis dysregulation exists.

Within studies, there was a high rate of inconsistency between clinical and cortisol findings, with cortisol findings supporting clinical findings in only 50 % of studies. In many cases, there was a significant clinical response to the intervention but no cortisol response. This may have occurred for a wide variety of reasons. The lack of cortisol response most likely reflects flaws in the cortisol measurement methodology, as discussed above. Lack of engagement of the HPA axis by the intervention is also a possibility, indicating that the intervention works by an alternative mechanism. Alternatively, it is possible that ‘target engagement’ did occur but that the impact on cortisol was obscured by the effects of other pathways and systems. Finally, another reason for lack of effect may be the absence of HPA axis dysregulation at baseline in the sample population receiving the intervention. For many of the studies, the prevalence or degree of HPA axis dysregulation in the population at baseline was not clear; this would need to be high in order to observe an improvement in HPA axis function after a therapeutic intervention, particularly in the presence of many confounders, as would be common in a patient population.

In a minority of cases of disagreement between findings, positive cortisol findings occurred in the absence of clinical findings. This may represent a time lag between HPA axis restoration and clinical improvement, with HPA axis restoration temporarily preceding clinical improvement. It may also result from the use of inappropriate clinical outcome measures, resulting in false negative clinical findings. Alternatively, however, this disagreement may reflect lack of reliability in the cortisol measure, resulting in false positive cortisol findings. Low reliability is highly likely for the studies included in this review, given the high prevalence of 1-day saliva collection protocols. Along with short-term reliability issues, the long-term stability of diurnal cortisol measures is also likely to impact on results, and there is a growing literature to suggest that this is low [17]. For example, Ross et al. [121] analysed visit-to-visit cortisol stability for the diurnal cortisol profile in a population of 46 healthy adults, providing 3-day cortisol profile samples at 2.5 monthly visits over 8 months and found only low-modest intra-class correlation coefficients (ICC) for the cortisol awakening response (ICC 0.219), the diurnal slope (ICC 0.473) and the area under the curve (ICC 0.556), with even lower stability at the individual level.

Due to heterogeneity across studies in relation to the HPA axis parameters measured, it was difficult to explore the timeframe over which a given parameter might be expected to change following an intervention, which was an important review aim. Nevertheless, the review has shed some light on this area of uncertainty by identifying that changes in parameters occurred at a median of 6 weeks from baseline (IQR 4–12). Though this finding needs to be interpreted with caution, given the wide heterogeneity across studies in relation to parameters used, intervention duration and follow-up schedule, it at least provides a guide for the design of future RCTs in relation to the optimal timing of the primary endpoint and the length of the follow-up period.

### Recommendations for the Future

In view of the increasing use of salivary diurnal cortisol as a biomarker within RCTs and the marked heterogeneity in practices and findings across studies, there is a clear need for guidance on how best to incorporate this biomarker into RCTs, in order to prevent unnecessary research costs and participant burden. The high level of inconsistency between clinical and cortisol findings and the difficulty in interpreting cortisol change patterns suggests a need for further validation studies. There is also a need for greater precision in diurnal cortisol measurement. Furthermore, there is a need for greater uniformity in the collection and analysis of cortisol, to allow findings to be compared across studies. We have summarised recommendations towards the achievement of these goals in box 1.

Box 1. Recommendations for the use of salivary diurnal cortisol as a biomarker within randomised controlled trials.

**Table:** 

A. Decide whether or not it will be a useful biomarker:
• Establish the prevalence and pattern of HPA axis dysregulation in the target population
• Establish the longitudinal change in the pattern of HPA axis activity over the planned time-frame for the RCT
• Establish the construct validity of HPA axis parameters against relevant clinical measures
• Be able to form an a priori hypothesis regarding the expected direction of change in at least one HPA axis parameter in response to the experimental intervention
B. Optimise the reliability and validity of the cortisol measure:
• Collect salivary cortisol over at least 2 days both before and at least once after the intervention
• Collect all samples with reference to awakening time rather than a clock time
• Ideally, include enough sample points in the day to analyse all three parameters (the cortisol awakening response, the diurnal slope and the area under the curve), to provide a full picture of HPA axis activity, unless there are valid reasons to exclude some components (e.g. expected high non-compliance rates for the cortisol awakening response)
C. Optimise the ability to interpret and compare clinical trial findings:
• Choose one cortisol parameter as the primary cortisol outcome measure (e.g. cortisol awakening response **or** area under the curve **or** diurnal slope) in advance of the study, linking this with the a priori hypothesis; this should be identified as the primary parameter in the protocol and the published report.
• Include all other cortisol parameters as secondary outcome measures

### Limitations

A number of methodological limitations need to be borne in mind when interpreting the findings of this review. Firstly, though we searched six electronic databases using sensitive search terms for RCTs and salivary cortisol, we excluded animal studies from three databases (MEDLINE, EMBASE and AMED) using the exploded term, which, we realised in retrospect, may have inadvertently eliminated some human studies. Having assessed the impact of this on the MEDLINE results, however, we are confident that this has not had a significant impact on the overall yield of eligible studies due to the substantial overlap of these databases with each other and with both the Cochrane Central Register of Controlled Trials and PsychINFO. Secondly, we did not perform a supplementary manual literature search. Whilst this strategy may have improved our yield of RCTs, given the very broad search criteria in relation to type of intervention and population, it was not feasible to devise a comprehensive manual search strategy without biasing the study selection process.

## Conclusions

This review systematically maps the literature which reports on the use of salivary diurnal cortisol as an outcome measure within RCTs. It demonstrates that there is wide heterogeneity across RCTs in the methodology of salivary cortisol collection, and in the profile parameters analysed. Furthermore, it has demonstrated that such methodological heterogeneity has consequences for both the internal validity of individual trials and the ability to compare and synthesise results across trials of similar interventions. As such, it highlights a need for better validation of this measure, more reliable approaches to measurement and the need for greater collaboration between the disciplines of psychoneuroendocrinology and applied science disciplines such as medicine, psychology and nursing, with a view to better and more prompt translation of basic science knowledge about HPA axis measurement into clinical trials research.

### Electronic Supplementary Material

Below is the link to the electronic supplementary material.

ESM 1 (DOCX 40 kb)

## Appendixes

### Appendix A

Search strategy for systematic review.

**Table:**

MEDLINE (1980 to 21 May 2015):
1. cortisol.ti,ab;
2. saliva*.af;
3.1 AND 2;
4. HYDROCORTISONE/;
5. SALIVA/;
6.4 AND 5;
7. ‘randomized controlled trial’.pt;
8. ‘controlled clinical trial’.pt;
9. ‘randomized’.ab;
10. placebo.ab;
11. randomly.ab;
12. trial.ab;
13. groups.ab;
14. 7 OR 8 OR 9 OR 10 OR 11 OR 12 OR 13;
15. expANIMALS/
16. 14 NOT 15
17. 3 OR 6
18. 16 AND 17;180 results
CINAHL (1981 to 21 May 2015):
1. cortisol.ti,ab;
2. saliva*".af;
3. HYDROCORTISONE/;
4. SALIVA/;
5. 1 AND 2;
6. 3 AND 4;
7. 5 OR 6;
8. ‘randomized controlled trial’.pt;
9. ‘controlled clinical trial’.pt;
10. ‘clinical trial’.pt;
11. RANDOMIZED CONTROLLED TRIALS/OR CLINICAL TRIALS/OR INTERVENTION TRIALS/;
12. randomized.ab;
13. placebo.ab;
14. randomly.ab;
15. trial.ab;
16. groups.ab;
17. 8 OR 9 OR 10 OR 11 OR 12 OR 13 OR 14 OR 15 OR 16;
18. 7 AND 17; 338 results
PsychINFO (1806 to 21 May 2015):
1 cortisol.ti,ab;
2. saliva*.af;
3. 1 AND 2;
4. HYDROCORTISONE/;
5. SALIVA/;
6. 4 AND 5;
7. 3 OR 6;
8. ‘randomised controlled trial’.pt
9. ‘controlled clinical trial’.pt
10. ‘clinical trial’.pt
11. TREATMENT EFFECTIVENESS EVALUATION/OR CLINICAL TRIALS/;
12. randomized.ab;
13. placebo.ab;
14. randomly.ab;
15. trial.ab;
16. groups.ab;
17. 8 OR 9 OR 10 OR 11 OR 12 OR 13 OR 14 OR 15 OR 16;
18. 7 AND 17; 879 results
AMED (1985 to 21 May 2015)
1 cortisol.ti,ab;
2. saliva*.af;
3. 1 AND 2;
4. HYDROCORTISONE/;
5. SALIVA/;
6. 4 AND 5;
8. ‘randomized controlled trial’.pt
9. ‘controlled clinical trial’.pt
10. ‘clinical trial’.pt
11. CLINICAL TRIALS/OR RANDOMIZED CONTROLLED TRIALS;
12. randomized.ab;
13. placebo.ab;
14. randomly.ab;
15. trial.ab;
16. groups.ab;
17. 8 OR 9 OR 10 OR 11 OR 12 OR 13 OR 14 OR 15 OR 16;
18. expANIMALS/
19. 17 NOT 18
20. 3 OR 6
21. 19 AND 20; 11 results
EMBASE (1974 to 21 May 2015)
1 cortisol.ti,ab;
2. saliva*.af;
3. 1 AND 2;
4. HYDROCORTISONE/;
5. SALIVA/OR SALIVA ANALYSIS/OR SALIVA COLLECTOR/
6. 4 AND 5
7. 3 OR 6
8. ‘CLINICAL TRIAL (topic)’/OR CONTROLLED CLINICAL TRIAL/OR ‘CONTROLLED CLINICAL TRIAL (topic)’/OR ‘PHASE 1 CLINICAL TRIAL (topic)’/OR ‘PHASE 2 CLINICAL TRIAL (topic)’/OR ‘PHASE 3 CLINICAL TRIAL (topic)’/OP ‘PHASE 4 CLINICAL TRIAL (topic)’/OR ‘RANDOMIZED CONTROLLED TRIAL (topic)’/
9. randomized.ab;
10. placebo.ab;
11. randomly.ab;
12. trial.ab;
13. groups.ab;
14. 8 OR 9 OR 10 OR 11 OR 12 OR 13
15. expANIMAL/
16. 14 NOT 15
17. 16 AND 7;109 results
Cochrane Central Register of Controlled Trials (up to 21 May 2015)
1. cortisol
2. saliva*
3. Mesh descriptor: hydrocortisone
4. Mesh descriptor: saliva
5. (1 AND 2) OR (3 AND 4); 857 results

### Appendix B

Quality and relevance assessment using Gough’s framework.

**Table:**

Study ID	Weight of Evidence A (Generic quality of execution of study)	Weight of Evidence B (Appropriateness of the study design to the review aim)	Weight of Evidence C (Focus of the study content relative to the review aim)	Weight of Evidence D (Overall quality and relevance grade)
Banasik et al. 2011 [86]	Low	High	High	Moderate
Barbadoro et al. 2013[60]	Moderate	High	Moderate	Moderate
Bergen-Cico et al. 2014 [22]	Moderate	High	High	High
Billhult et al. 2008 [87]	Moderate	High	Low	Moderate
Boelens et al. 2009 [104]	Low	High	Moderate	Moderate
Bormann et al. 2009 [23]	Moderate	High	Moderate	Moderate
Bougea et al. 2013 [24]	Low	High	Moderate	Moderate
Bower et al. 2014 [88]	Moderate	High	High	High
Camfield et al. 2013 [61]	Low	High	High	Moderate
Campo et al. 2015 [90]	Low	High	High	Moderate
Carlson et al. 2013 [25]	High	High	High	High
Cash et al. 2014 [26]	Moderate	High	High	High
Chaborski et al. 2015 [62]	Moderate	High	Moderate	Moderate
Chan et al. 2006 [27]	Moderate	High	Moderate	Moderate
Chandwani et al. 2014 [92]	Moderate	High	High	High
Chen et al. 2013 [93]	Moderate	High	High	High
Corey et al. 2014 [94]	Moderate	High	High	High
Danucalov et al. 2013 [96]	Moderate	High	Moderate	Moderate
Delle Chiaie et al. 2012 [28]	Low	High	High	Moderate
Deuschle et al. 2003 [63]	Moderate	Low	Moderate	Moderate
Dudgeon et al. 2012 [105]	Low	High	Moderate	Moderate
Eijsbouts et al. 2008 [64]	Moderate	High	Moderate	Moderate
Emery et al. 2005 [106]	Low	High	Moderate	Moderate
Feicht al. 2013 [29]	Low	High	Moderate	Moderate
Gaab et al. 2006 [30]	Moderate	High	High	High
Garrison and Chambliss, 2006 [65] & Kalman et al. 2008 [66]	Low	High	Moderate	Moderate
Gex-Fabry et al. 2012 [31]	High	High	High	High
Hellweg et al. 2008 [67]	Moderate	High	Moderate	Moderate
Hinkelmann et al. 2012 [68]	Moderate	Moderate	High	Moderate
Hodgson and Lafferty, 2012 [97]	Moderate	High	Moderate	Moderate
Holt-Lunstad et al. 2008 [32]	Moderate	High	Moderate	Moderate
Hsiao et al. 2011 [33]	Moderate	High	Moderate	Moderate
Hsiao et al. 2012 [34]	Low	High	High	Moderate
Hsiao et al. 2014 [35]	Low	High	High	Moderate
Huang et al. 2012 [98]	Low	High	High	Moderate
Jaggers et al. 2014 [107]	Low	High	Moderate	Moderate
Jensen et al. 2012 [36]	Moderate	High	Low	Moderate
Klatt et al. 2009 [37]	Low	High	Moderate	Moderate
Knorr et al. 2012 [70]	High	High	High	High
Krajewski et al. 2010 [38]	Low	Moderate	Moderate	Moderate
Lenze et al. 2011 [71] & Lenze et al. 2012 [72]	Low	High	Moderate	Moderate
Letourneau et al. 2011 [39]	Low	High	Moderate	Moderate
Lindh-Astrand et al. 2013 [41]	Moderate	High	Moderate	Moderate
Lipschitz et al. 2013 [42]	Moderate	High	High	High
Lieverse et al. 2011 [108]	High	High	Moderate	High
Limm et al. 2011 [40]	High	High	Moderate	High
Lok et al. 2012 [44]	High	High	Moderate	High
Lopresti et al. 2015 [73]	Moderate	High	Moderate	Moderate
Mocking et al. 2014 [75]	Moderate	High	Moderate	Moderate
Nickel, C et al. 2007 [46] & Nickel, M.K., 2007 [47]	Moderate	High	Moderate	Moderate
Nonino-Borges et al., 2007 [76]	Moderate	High	Moderate	Moderate
Nunes et al., 2007 [48]	Moderate	High	High	High
Oken et al. 2010 [49]	Moderate	High	Moderate	Moderate
Pacella et al. 2014 [50]	Moderate	Moderate	Moderate	Moderate
Plag et al. 2014 [51]	Moderate	High	Moderate	Moderate
Raghavendra et al. 2009 [99]	Low	High	High	Moderate
Richter et al. 2012 [53]	Low	High	High	Moderate
Ruhe et al. 2015 [77]	Moderate	Moderate	Moderate	Moderate
Saxton et al. 2014 [109]	Moderate	High	High	High
Scharnholz et al. 2010 [78]	Moderate	High	Moderate	Moderate
Scherder et al. 2003 [111]	Moderate	High	High	High
Scherder et al. 2006 [112]	Moderate	High	High	High
Schmidt et al. 2015 [79]	Moderate	High	High	High
Schneider et al. 2007 [100]	Low	High	High	Moderate
Schubert et al., 2011 [80]	Moderate	High	Moderate	Moderate
Sears et al., 2007 [54]	Low	High	Moderate	Moderate
Talbott et al. 2013a [81]	Low	High	Moderate	Moderate
Talbott et al. 2013b [82]	Low	High	High	Moderate
Tam et al. 2014 [113]	Moderate	High	Moderate	Moderate
Taylor et al., 2009 [55]	Moderate	High	High	High
Tornhage et al. 2013 [102]	Low	Moderate	Moderate	Moderate
Tucker et al. 2004 [83]	Moderate	High	Moderate	Moderate
Urizar and Munoz, 2011 [56]	Moderate	High	Moderate	Moderate
Walsh et al. 2006 [84]	Moderate	Moderate	Moderate	Moderate
Wilcox et al. 2014 [57]	Moderate	High	Moderate	Moderate
Witbracht et al. 2013 [85]	Moderate	High	Moderate	Moderate
Woods et al. 2009[103]	Low	Moderate	Moderate	Moderate
Yang et al. 2009 [59]	Low	High	High	Moderate
