# Supplementary material for: Use of Salivary Diurnal Cortisol as an Outcome Measure in Randomised Controlled Trials: a Systematic Review
Source: Ann Behav Med. 2016 Mar 23;50:210–36. doi: 10.1007/s12160-015-9753-9 (PMC4823366; doi:10.1007/s12160-015-9753-9)
Supplement: Supplementary file 1 — (DOCX 40 kb) [file 12160_2015_9753_MOESM1_ESM.docx]

**Protocol Title:**

**Use of salivary diurnal cortisol as an outcome measure within randomised controlled trials**

**Background:**

The hypothalamic-pituitary-adrenal (HPA) axis is known to be an important pathway in the regulation of the physiological stress response. HPA axis dysregulation has been shown to be associated with important health outcomes including psychiatric illness (1), cardiovascular mortality (2), cancer prognosis (3,4), frailty and cognitive decline (5). These associations are thought to be mediated by the deleterious effects of chronic stress on HPA axis function (6), with secondary effects on metabolic, immune and psychobiological systems (7). The many associations between HPA axis dysregulation and markers of health status suggest that the modulation of the HPA axis by interventions may have a role in disease treatment and prevention.

The measurement of diurnal patterns of cortisol secretion in blood or saliva is an established method of measuring HPA axis function (8) . Typically, under basal conditions, a healthy HPA axis is characterised by a distinctive circadian pattern of cortisol secretion whereby cortisol rises to a peak within 30-45 minutes of waking and then falls to a nadir at approximately midnight (8). This pattern becomes disrupted when the HPA axis becomes dysregulated, with loss of normal rhythm and responsiveness (9,10). The pattern of cortisol profile disruption appears to vary depending on the context or condition studied but, in general, an abnormal cortisol awakening response (CAR) or a flattened diurnal cortisol slope appear to be consistent markers of HPA axis dysfunction (8).

The use of salivary cortisol as a biomarker is not a new concept and is a well-established practice in stress research (11). There has been wide variation in the salivary cortisol measurement conditions and methods used within interventional studies, however. Most studies do not appear to measure diurnal cortisol profiles and instead measure a single cortisol level or the response of cortisol to a stress task. Single basal cortisol values measured at a fixed time are known to be associated with significant intra-individual variability as well as inter-individual variability (12), thus yielding inconsistent results. The response of cortisol to a stress-task reflects simulated cortisol rather than basal cortisol secretory function and is, therefore, not a reflection of basal HPA axis function. Those studies which do measure diurnal salivary cortisol profiles appear to use different collection and analytic methods, rendering comparison and interpretation challenging.

As diurnal cortisol profile patterns have been linked with important health outcomes, this type of salivary cortisol measurement would appear to be the more pertinent outcome measure for evaluating potential HPA-axis-modulating interventions. Little is known about the use of this type of measurement for this purpose, however. Due to the vast number of studies using salivary cortisol as an outcome measure in a wide variety of different ways, it is not readily apparent from the literature which interventions have been evaluated using this specific method and which particular methodological approaches have been used therein. In addition, though HPA axis plasticity is thought to be scientifically plausible, it is not known whether salivary diurnal cortisol profile measurement is the correct tool for measuring HPA axis change as little is known about its responsiveness as a research outcome measure. Furthermore, it is thought that different parts of the diurnal cortisol profile are separately regulated by the HPA axis, suggesting that some parts of the HPA axis may be more plastic than others. It is not clear which diurnal cortisol profile parameters are more amenable to change, however.

**Objectives:**

We aim to systematically review the randomised controlled trials in the literature which have used diurnal salivary cortisol as an outcome measure in order to describe how this measure has been used thus far for evaluating interventions. We hope that this descriptive review will enable us to make judgments about the responsiveness of the measure and the parameters of the measure which appear to be most useful within clinical trials. We seek to answer the following questions:

1. Which interventions have been evaluated using diurnal salivary cortisol profile measures?
2. Which methods have been used to collect salivary cortisol profile measures?
3. Which parameters of the salivary cortisol profile have been used in the analysis of intervention efficacy?
4. Which interventions have shown a significant change in a salivary cortisol profile parameter and when?
5. What is the consistency between cortisol and clinical outcomes?

**Methods:**

***Study Inclusion Criteria (in order of importance):***

1. Study Design: randomised controlled trials
2. Outcome measures: studies that use diurnal salivary cortisol profile measurement as a primary or secondary outcome measure
3. Types interventions: any intervention which is used as a therapy, excluding exogenous corticosteroids
4. Controls: any type of control
5. Population: any human adult (>18 years) population

***Study Exclusion Criteria:***

1. Non-RCT studies: quasi- randomised controlled trials (including alternate allocation), observational and descriptive studies, review articles, opinion articles, trial protocol reports without results
2. Studies which use other non-diurnal salivary sampling methods e.g. single salivary cortisol measures pre and post an intervention or salivary cortisol pre and post a stress-task
3. Studies which evaluate the effects of exogenous steroids (any type or route) on salivary cortisol
4. Studies which evaluate the cortisol response to stress-inducing interventions as opposed to therapeutic interventions
5. Studies which measure diurnal cortisol under laboratory-induced conditions (e.g. light-wake conditions)
6. Animal studies
7. Abstract publication available only
8. Dissertation available only
9. Non-English language publications
10. Studies including patients with Cushings Disease

***Definitions:***

According to Adam et al. (8) the diurnal rhythm of salivary cortisol secretion can be calculated by calculating the slope between a minimum of two salivary cortisol collection points over a 1 day period (e.g. one morning and one evening sample). Therefore, it is possible to calculate at least one parameter of the diurnal cortisol profile using two sample collection points. Based on this rationale, we have defined salivary diurnal cortisol profile measurement as the collection of at least two samples of cortisol over at least 1 day. Within a randomised controlled trial investigating an intervention there will need to be evidence that this procedure has been conducted on at least one occasion before the intervention and on at least one separate occasion (i.e. a separate day or period of days) after the intervention. If the before- and after- cortisol levels are collected on the same day, this does not constitute a diurnal cortisol profile.

***Search methods for identification of studies:***

1. Electronic searches:

Six electronic databases will be searched: MEDLINE, EMBASE,Cochrane Central Register for Controlled Trials, AMED, PSYCHINFO, CINAHL

See appendix for detailed search strategies.

***Data collection and analysis:***

1. Selection of studies:

All abstracts generated from the electronic searches will be exported to Endnote for removal of duplicates. One review author (RR) will then screen the titles and abstracts for the eligibility criteria and will record the reasons for exclusion on an Excel spreadsheet. When RR cannot determine eligibility from the abstract or where the abstract suggests potential eligibility, the full text article will be obtained. RR will divide up a selection of the full text articles between 4 other review authors (SB, AS, AC, SM) and distribute them for review. RR will review all of the full text articles independently herself. Each of the other authors will review their assigned portion of the full text articles. In this way, a proportion of the articles will have had independent duplicate review (proportion will depend on full text article yield and time constraints). Any disagreement will be discussed in the first instance between the two authors in question. If consensus cannot be achieved between the two authors in question, a third party (one of the other authors) will be consulted. If consensus cannot be achieved at this point, further information will be sought until a decision can be made. Trial flow will be described in a trial flow diagram according to the PRISMA criteria.

1. Data extraction:

All full text articles which have been deemed eligible will then be reviewed in more detail for data extraction. This will be performed by RR. 10% of the full text articles will be reviewed for data extraction by another author to check that RR is doing it appropriately. A data extraction form containing the following fields will be used to summarise the pertinent details of the study: Trial ID, Eligibility Criteria checklist with decision outcome, Study design, Population, Intervention/Control, Salivary Cortisol Collection Details, Other outcome measures, Salivary Cortisol Analysis Details, Salivary Cortisol Results.

We aim to describe the breadth of variation in utilisation of salivary cortisol so we do not wish to restrict our data extraction to predetermined data criteria. All forms of analysis and result reporting will be recorded and used.

1. Assessment of quality and relevance of included studies:

For each included full-text article, RR will assess quality and relevance using The Gough Framework.

1. Assessment of heterogeneity:

We expect that there will be significant clinical and methodological heterogeneity in this study. We will simply assess this by inspecting the situations, populations and methodological approaches across the studies. We will not test for statistical heterogeneity, given the expected high degree of heterogeneity.

***Data synthesis:***

Due to the expected heterogeneity in the selected studies, we do not intend to do a quantitative data synthesis where the results of the studies are combined. We will simply tabulate the characteristics and results of each study and do a narrative synthesis of the findings in relation to each research question. Within this approach we will count the following in relation to salivary cortisol:

1. Type and frequency of interventions and controls evaluated using salivary diurnal cortisol profiles
2. Type and frequency of different collection schedules (number of days of collection and number of collection time-points per day)
3. Type and frequency of different salivary diurnal cortisol profile parameters measured
4. Number of studies in which salivary diurnal cortisol profile is a primary outcome measure
5. Number of studies which demonstrate a statistically significant change in any parameter of the salivary diurnal cortisol profile on comparison of intervention and control groups
6. Number of studies which detect a change in both cortisol and the clinical outcome measures, percentage of studies showing a change in neither, percentage of studies with conflicting results between salivary cortisol and the clinical outcome measures.

If possible, if a significant change has been detected in a salivary cortisol parameter, we will correlate that change with the change in the primary clinical outcome measure in order to assess responsiveness.

**References:**

1. De Kloet ER, Joëls M, Holsboer F. Stress and the brain: from adaptation to disease. Nature reviews. Neuroscience [Internet]. 2005 Jun [cited 2013 Aug 6];6(6):463–75. Available from: http://www.ncbi.nlm.nih.gov/pubmed/15891777

2. Kumari M, Shipley M, Stafford M, Kivimaki M. Association of diurnal patterns in salivary cortisol with all-cause and cardiovascular mortality: Findings from the Whitehall II study. Journal of Clinical Endocrinology and Metabolism. 2011;96(5):1478–85.

3. Sephton SE, Sapolsky RM, Kraemer HC, Spiegel D. Diurnal cortisol rhythm as a predictor of breast cancer survival. Journal of the National Cancer Institute [Internet]. 2000 Jun 21;92(12):994–1000. Available from: http://www.ncbi.nlm.nih.gov/pubmed/10861311

4. Sephton SE, Lush E, Dedert EA, Floyd AR, Rebholz WN, Dhabhar FS, et al. Diurnal cortisol rhythm as a predictor of lung cancer survival. Brain, Behavior, and Immunity. 2013;30(SUPPL):S163–S170.

5. Kumari M, Badrick E, Sacker A, Kirschbaum C, Marmot M, Chandola T. Identifying patterns in cortisol secretion in an older population. Findings from the Whitehall II study. Psychoneuroendocrinology [Internet]. Elsevier Ltd; 2010 Aug [cited 2013 Aug 15];35(7):1091–9. Available from: http://www.ncbi.nlm.nih.gov/pubmed/20171018

6. Fries E, Hesse J, Hellhammer J, Hellhammer DH. A new view on hypocortisolism. Psychoneuroendocrinology. 2005;30(10):1010–6.

7. McEwen BS. The neurobiology of stress: from serendipity to clinical relevance. Brain Research. 2000;886(1-2):172–89.

8. Adam EK, Kumari M. Assessing salivary cortisol in large-scale, epidemiological research. Psychoneuroendocrinology [Internet]. 2009 Nov [cited 2013 Aug 15];34(10):1423–36. Available from: http://www.ncbi.nlm.nih.gov/pubmed/19647372

9. Clow A. Cortisol as a biomarker of stress . Journal of holistic healthcare. 2004;1(3):10–4.

10. De Kloet ER. Hormones and the stressed brain. Annals of the New York Academy of Sciences. 2004;1–15.

11. Hellhammer DH, Wüst S, Kudielka BM. Salivary cortisol as a biomarker in stress research. Psychoneuroendocrinology [Internet]. 2009 Feb [cited 2013 Aug 12];34(2):163–71. Available from: http://www.ncbi.nlm.nih.gov/pubmed/19095358

12. Wüst S, Federenko I, Hellhammer DH, Kirschbaum C. Genetic factors, perceived chronic stress, and the free cortisol response to awakening. Psychoneuroendocrinology [Internet]. 2000 Oct;25(7):707–20. Available from: http://www.ncbi.nlm.nih.gov/pubmed/10938450

**APPENDIX:**

**MEDLINE:**

1. cortisol.ti,ab;

2. saliva*.af;

3.1 AND 2;

4. HYDROCORTISONE/;

5. SALIVA/;

6.4 AND 5;

7. “randomized controlled trial”.pt;

8. “controlled clinical trial”.pt;

9. “randomized”.ab;

10. placebo.ab;

11. randomly.ab;

12. trial.ab;

13. groups.ab;

14. 7 OR 8 OR 9 OR 10 OR 11 OR 12 OR 13;

15. expANIMALS/

16. 14 NOT 15

17. 3 OR 6

18. 16 AND 17;180 results

**CINAHL:**

1. cortisol.ti,ab;

2. saliva*".af;

3. HYDROCORTISONE/;

4. SALIVA/;

5. 1 AND 2;

6. 3 AND 4;

7. 5 OR 6;

8. “randomized controlled trial”.pt;

9. “controlled clinical trial”.pt;

10. “clinical trial”.pt;

11. RANDOMIZED CONTROLLED TRIALS/OR CLINICAL TRIALS/OR INTERVENTION TRIALS/;

12. randomized.ab;

13. placebo.ab;

14. randomly.ab;

15. trial.ab;

16. groups.ab;

17. 8 OR 9 OR 10 OR 11 OR 12 OR 13 OR 14 OR 15 OR 16;

18. 7 AND 17; 338 results

**PsychINFO:**

1 cortisol.ti,ab;

2. saliva*.af;

3. 1 AND 2;

4. HYDROCORTISONE/;

5. SALIVA/;

6. 4 AND 5;

7. 3 OR 6;

8. “randomised controlled trial”.pt

9. “controlled clinical trial”.pt

10. “clinical trial”.pt

11. TREATMENT EFFECTIVENESS EVALUATION/OR CLINICAL TRIALS/;

12. randomized.ab;

13. placebo.ab;

14. randomly.ab;

15. trial.ab;

16. groups.ab;

17. 8 OR 9 OR 10 OR 11 OR 12 OR 13 OR 14 OR 15 OR 16;

18. 7 AND 17; 879 results

**AMED**

1 cortisol.ti,ab;

2. saliva*.af;

3. 1 AND 2;

4. HYDROCORTISONE/;

5. SALIVA/;

6. 4 AND 5;

8. “randomized controlled trial”.pt

9. “controlled clinical trial”.pt

10. “clinical trial”.pt

11. CLINICAL TRIALS/OR RANDOMIZED CONTROLLED TRIALS;

12. randomized.ab;

13. placebo.ab;

14. randomly.ab;

15. trial.ab;

16. groups.ab;

17. 8 OR 9 OR 10 OR 11 OR 12 OR 13 OR 14 OR 15 OR 16;

18. expANIMALS/

19. 17 NOT 18

20. 3 OR 6

21. 19 AND 20; 11 results

**EMBASE**

1 cortisol.ti,ab;

2. saliva*.af;

3. 1 AND 2;

4. HYDROCORTISONE/;

5. SALIVA/OR SALIVA ANALYSIS/OR SALIVA COLLECTOR/

6. 4 AND 5

7. 3 OR 6

8. “CLINICAL TRIAL (topic)”/OR CONTROLLED CLINICAL TRIAL/OR “CONTROLLED CLINICAL TRIAL (topic)”/OR “PHASE 1 CLINICAL TRIAL (topic)”/OR “PHASE 2 CLINICAL TRIAL (topic)”/OR “PHASE 3 CLINICAL TRIAL (topic)”/OP “PHASE 4 CLINICAL TRIAL (topic)”/OR “RANDOMIZED CONTROLLED TRIAL (topic)”/

9. randomized.ab;

10. placebo.ab;

11. randomly.ab;

12. trial.ab;

13. groups.ab;

14. 8 OR 9 OR 10 OR 11 OR 12 OR 13

15. expANIMAL/

16. 14 NOT 15

17. 16 AND 7;109 results

**Cochrane Central Register of Controlled Trials**

1. cortisol

2. saliva*

3. Mesh descriptor: hydrocortisone

4. Mesh descriptor: saliva

5. (1 AND 2) OR (3 AND 4); 857 results
